# ALDH2 Overexpression Alleviates High Glucose-Induced Cardiotoxicity by Inhibiting NLRP3 Inflammasome Activation

**DOI:** 10.1155/2019/4857921

**Published:** 2019-11-21

**Authors:** Ruiping Cao, Dian Fang, Jiahui Wang, Ying Yu, Hongwei Ye, Pinfang Kang, Zhenghong Li, Hongju Wang, Qin Gao

**Affiliations:** ^1^Department of Physiology, Bengbu, Anhui 233030, China; ^2^Department of Cardiovascular Disease, The First Affiliated Hospital of Bengbu Medical College, Bengbu, Anhui 233004, China

## Abstract

Although the underlying mechanisms of diabetes-induced myocardial injury have not been fully illuminated, the inflammation reaction has been reported intently linked with diabetes. The nucleotide binding oligomerization domain-like receptor protein 3 (NLRP3) inflammasome, the key component of pyroptosis, is involved in inflammation reaction, which may be one of the important mechanisms in diabetes-induced myocardial injury. The purpose of this study was to investigate the changes of NLRP3 inflammasome and pyroptosis in high glucose-induced H9C2 cardiac cell injury and investigate whether overexpression of mitochondrial aldehyde dehydrogenase 2 (ALDH2) can reduce the occurrence of pyroptosis. The H9C2 cardiac cells were exposed to 35 mM glucose for 24 h to induce cytotoxicity. Mitochondrial ALDH2 overexpression cardiac cell line was constructed. The results showed in high glucose condition that ALDH2 overexpression significantly increased H9C2 cardiac cell viability, increased mitochondrial ALDH2 activity and protein expression, and reduced mitochondrial reactive oxygen species (ROS) production, 4-hydroxynonenal (4-HNE), and lactate dehydrogenase (LDH) levels; meanwhile, the pyroptosis key components—NLRP3 inflammasome-related proteins, apoptosis-associated speck-like protein containing a caspase recruitment domain (ASC), cysteine-containing aspartate specific protease 1 (Caspase-1), and interleukin-18 (IL-18) protein expressions—were significantly decreased, and IL-18 and interleukin-1*β* (IL-1*β*) levels were also decreased. In high glucose-induced cardiac cell injury, ALDH2 overexpression may reduce ROS production, thereby inhibiting the activation of NLRP3 inflammation and cell pyroptosis. ALDH2 gene might play the potential role in the treatment of high glucose-induced H9C2 cardiac cell injury.

## 1. Introduction

Diabetes is associated with abnormal cardiac structure and function; cardiovascular complications remain the major cause of mortality and morbidity in diabetic patients [1]. Diabetic cardiomyopathy (DCM) is characterized by cardiac structural and functional impairment, including myocardial fibroblast activation, left ventricular dysfunction, cardiac cell death, and metabolic disorders. Among them, the cardiac cell death and cardiac fibrosis are considered the fundamental changes in DCM, which can cause cardiac remodeling and accelerate DCM development, which eventually leads to heart failure [2]. There are many patterns of cell death, including apoptosis, autophagy, necroptosis, and pyroptosis. In recent years, as an important cell death closely related to inflammatory response, pyroptosis has attracted scientists' attention.

Pyroptosis is a highly regulated process of cell death, which is essential for physiological processes such as organ development, cell renewal, and differentiation [3]. Compared with the classical cell death patterns such as apoptosis, pyroptosis is Caspase-1-dependent cell death accompanied with a large number of inflammatory cytokine releases [4]. Pyroptosis is characterized by rapid destruction of the plasma membrane and loss of membrane integrity; causes extracellular water influx, cell osmotic lysis, and cellular proinflammatory mediator release including interleukin-18 (IL-18) and interleukin-1*β* (IL-1*β*); and then leads to cascade amplification of inflammatory responses, finally promoting the development of diseases [5]. It is well known that inflammatory response is involved in the development of cardiovascular diseases and pyroptosis may play a key role in the pathogenesis of cardiovascular diseases [6].

Inflammasome as an important product of inflammatory reaction is associated closely with pyroptosis. There are many types of inflammasome; the nucleotide-binding oligomerization domain-like receptor protein 3 (NLRP3) inflammasome is one of the most widely studied [6], which is mainly composed of NLRP3 protein, apoptosis-associated speck-like protein containing a caspase recruitment domain (ASC), and Caspase-1 [7, 8]. Activation of NLRP3 inflammasome plays an important role in pyroptosis. Hyperglycemia as the major feature of diabetes can activate a series of pathological molecular pathways, leading to excessive production of reactive oxygen species (ROS), inflammatory cell aggregation, excessive adhesion molecules release, and excessive ROS production meanwhile stimulating a variety of inflammatory factors release and induce inflammatory reactions [9]. Especially, in diabetes, hyperglycemia can induce mitochondrial ROS overproduction and mitochondria dysfunction, which may mediate inflammatory responses. Several studies have confirmed that mitochondrial ROS is necessary for the activation of NLRP3 inflammasome [10, 11]. However, whether reducing mitochondrial ROS release can reduce the occurrence of pyroptosis in DCM had few reports. Does the inflammation exist persistently with pyroptosis closely during diabetes course? It arouses our research interest.

Mitochondrial acetaldehyde dehydrogenase 2 (ALDH2) is a nuclear-coded aldehyde oxidase located in the mitochondrial matrix. A large number of studies had confirmed that ALDH2 could decompose acetaldehyde metabolite 4-hydroxynonenal (4-HNE), mitigating oxidative damage to cells by acetaldehyde and its metabolites [12]. ALDH2 has protective effects on the myocardium such as myocardial ischemia/reperfusion injury, alcohol-induced myocardial injury, and diabetes [13, 14]. Inhibition of ALDH2 potentiates high glucose-induced cardiomyocyte injury [15]. But what is the link between ALDH2 and NLRP3 inflammasome in cardiac cells? Is there a close relationship with mitochondrial oxidative stress? Whether activating ALDH2 expression in cardiac cells can inhibit the occurrence of pyroptosis has not been reported.

So in the study, we speculate that high glucose intervention can induce NLRP3 inflammasome formation and pyroptosis occurrence in a H9C2 cardiac cell model. ALDH2 can attenuate high glucose-induced cardiac injury by suppressing mitochondrial ROS production and inhibiting NLRP3 inflammasome activation.

## 2. Materials and Methods

### 2.1. Cell Lines and Transfection

The H9C2 cells were obtained from Shanghai GeneChem Co., Ltd. (Shanghai, China). The cell lines were maintained in Dulbecco's modified Eagle's medium (DMEM, HyClone, USA) with 10% fetal bovine serum (FBS, HyClone, USA) and 100 units/ml penicillin (Beyotime Biotechnology, Shanghai, China) at 37°C in a 5% CO_2_ humidified atmosphere.

### 2.2. Cell Transfection: Establishment of the ALDH2 Stable Overexpression and Negative Control Cell Lines

To establish the stable transduction, the lentiviral vectors expressing ALDH2 sequence, target gene virus, and the negative control virus were obtained from Shanghai GeneChem Co., Ltd. (Shanghai, China). Polybrene (Cat No. REVG0001, Shanghai, China) was used to promote the transfection according to the manufacturer's instruction.

#### 2.2.1. Determination of the Concentration of Puromycin in H9C2 Cells

Puromycin is a protein synthesis inhibitor and commonly used as the resistance screening reagent for stable transfection. Because if the lentivirus carries the puromycin resistance gene, when the virus is successfully infected, H9C2 cell line which obtains the puromycin resistance gene will not be killed by puromycin. Therefore, before screening for cells after infection with puromycin, the optimal screening concentration of puromycin should be determined first for the next experiments, and the concentration of puromycin that killed the normal cells within 72 hours is selected. The preliminary experiment is established that the minimum effective concentration of puromycin was 1 *μ*g/ml.

#### 2.2.2. Lentivirus Transfection

The procedure was performed according to the manufacturer's instructions. On a previous day, a total of 1 × 10^5^ cells were seeded per well in a 6-well culture plate and grown to 20-40% confluence. Virus and infection-enhancing fluid mixture were prepared according to the instructions and were added directly to the cells. Then, the cells were transfected at a multiplicity of infection (MOI) of 40 for 12 h.

A selection procedure was then conducted for another 72 h, by treating transduced cells with DMEM medium containing 1 *μ*g/ml puromycin (Beyotime Biotechnology, Shanghai, China). The transfection efficiency was observed using a fluorescence microscope. After that, all cells were passaged for three times for further biochemical assessments. The ALDH2 mRNA level was detected by real-time quantitative PCR, and the protein level was determined by western blot. After screening, H9C2 cardiac cells stably overexpressing ALDH2 gene (ALDH2-GFP cells) and negative control (GFP cells) were established.

### 2.3. Experimental Grouping

The cells were cultured in a 25 cm^2^ culture flask. When the cells were covered more than 70%, the serum-free medium was given for 24 h, and the experiments were divided into 6 groups as follows:

1. Group 1: the normal control group (NG)—the H9C2 cardiac cells were cultured in DMEM medium with 25 mM glucose (in which the basal culture medium for H9C2 cardiac cells is 25 mM) [16]
2. Group 2: the lentivirus negative control group (GFP)—the GFP transfer cells were cultured with 25 mM glucose. This group was to exclude the effect of the transfection on cells
3. Group 3: the ALDH2 overexpression group (ALDH2-GFP)—the ALDH2-GFP cells were cultured with 25 mM glucose
4. Group 4: the high-glucose group (HG)—the H9C2 cardiac cells were cultured with 35 mM glucose for 24 h to induce cell injury
5. Group 5: the high-glucose+GFP group (HG+GFP)—the GFP transfer cells were cultured with 35 mM glucose for 24 h
6. Group 6: the high-glucose+ALDH2-GFP group (HG+ALDH2-GFP)—the ALDH2-GFP cells were cultured with 35 mM glucose for 24 h

### 2.4. Cell Viability Measurement by CCK-8 Method

Cell viability was determined by CCK-8 Assay Kit (Biosharp, Hefei, China). H9C2 cardiac cells were cultured and treated in 96-well plates; 10 *μ*l CCK-8 reagent was added to each well followed by 2 h incubation. Absorbance was measured at 450 nm with a microplate reader (BioTek Instruments, Inc., Winooski, VT, USA). The mean optical density (OD) at 490 nm was used to calculate the percent of cell viability with the following formula:

(1) $$\begin{array}{c} \text{Cell viability }\left(\%\right)=\frac{\left(\text{dosing cell OD}-\text{blank OD}\right)}{\left(\text{control cell OD}-\text{blank OD}\right)\times 100}. \end{array}$$

### 2.5. Mitochondrial Oxidative Stress Measurement by Mitosox Staining Method

The H9C2 cardiac cells were cultured at a density of 1 × 10^5^/cells/ml. The cells were treated with 35 mM glucose for 24 h in the CO_2_ incubator at 37°C. Mitosox (Thermo Fisher Scientific, USA) was added to the final concentration at 5 *μ*M in the medium according to the manufacturer's recommendation. After 10 min, the cells were washed 2 times with Hank's Buffered Salt Solution (Gibco, USA) containing calcium and magnesium before measurement. The fluorescent image and intensity of Mitosox were measured at 510 nm (excitation wavelength) and 580 nm (emission wavelength) by fluorescent microscopy (FV-1200MPE SHARE, Olympus Japan). The ratio of cell fluorescent intensities of Mitosox to the fluorescent beads was calculated.

### 2.6. Lactate Dehydrogenase (LDH) Measurement in Supernatant

The lactate dehydrogenase (LDH) level in cellular supernatants was determined using LDH assay kit (Jiancheng, Nanjing, China) according to the manufacturer's instructions. The absorbance was measured at 450 nm.

### 2.7. Mitochondrial ALDH2 Activity Assay

The mitochondrial ALDH2 activity in cell lysates was detected by mitochondrial ALDH2 activity assay kit (Abcam, Cambridge, UK) according to the manufacturer's instructions. This assay kit is based on the ability of ALDH2 to catalyse the substrate NAD^+^ to produce NADH, which has strong absorption at 450 nm.

### 2.8. The Measurement of 4-HNE, IL-1*β*, and IL-18 Levels in Supernatant by ELISA Method

The levels of 4-HNE, IL-1*β*, and IL-18 in cell supernatants were examined by ELISA kits (Dakewe Biotech Co., Ltd., Shenzhen, China) according to the manufacturer's instructions. Briefly, a total of 1 × 10^6^/cells were cultured for 16~18 h, and then the serum-free medium was given for 24 hours. After high glucose intervention, the supernatant was centrifuged at 2000 r/min for 10 min. The absorbance (OD value) was measured at 450 nm.

### 2.9. ALDH2 mRNA Measurement by Real-Time Quantitative PCR (RT-PCR)

Total RNA was extracted from H9C2 cardiac cells using TRIzol® reagent (Invitrogen; Thermo Fisher Scientific, USA); then the total RNA (3 *μ*g) was transcribed to cDNA with RT reagent kit (Thermo Fisher Scientific, USA) at 42°C for 60 min and 70°C for 5 min. The sequences for ALDH2 primers were as follows: forward 5′- GTG TTC GGA GAC GTC AAA GA -3′ and reverse 5′-GCA GAG CTT GGG ACA GGT AA-3′ for ALDH2; forward 5′-ACA GCA ACA GGG TGG TGG AC-3′ and reverse 5′-TTT GAG GGT GCA GCG AAC TT-3′ for GAPDH. The ALDH2 expression level was quantified using 20 *μ*l reaction mixture containing 2 *μ*g cDNA, 0.3 mmol/L of each primer, and 10 *μ*l SYBR Green in 96-well plates. RT-PCR amplification conditions were as follows: 95°C for 30 sec, 40 cycles at 95°C for 5 sec and 60°C for 30 sec, and 72°C for 35 sec, followed by a final extension step at 72°C for 10 min, and the relative expression was analyzed by the 2^-*ΔΔ*Cq^ value calculation method and standardized against the housekeeping gene GAPDH.

### 2.10. ALDH2, NLRP3, ASC, Caspase-1, IL-18, and Caspase-3 Protein Expressions by Western Blot Method

The H9C2 cardiac cells were collected; after being washed twice with cold PBS buffer, the samples were lysed in a RIPA buffer (Beyotime Biotechnology, Shanghai, China) and then centrifuged (12000 rpm, 4°C) for 15 min. The supernatants were collected, and the concentration was measured using a BCA protein assay kit (Beyotime Biotechnology, Shanghai, China). Afterwards, the total protein samples were mixed with 5 times loading dye buffer, then heated at 95°C for 5 min. Total protein (30 *μ*g from each sample) was loaded on 10% sodium dodecyl sulfate-polyacrylamide gel electrophoresis (SDS-PAGE) and transferred to a polyvinylidene difluoride membrane, then subsequently blocked with 5% free-fat milk in TBST buffer for 2 h at room temperature.

The primary antibodies against ALDH2 (1 : 3000, Abcam, UK), NLRP3 (1 : 500, Novus, USA), ASC (1 : 500, Novus, USA), Caspase-1 (1 : 500, Abcam, UK), IL-18 (1 : 2000, R&D, USA), Caspase-3 (1 : 500, Novus, USA), *β*-actin (1 : 1000, Multi Sciences, China), and GAPDH (1 : 3000, Absin, China) were used in the experiments. The membranes were treated with appropriate primary antibodies overnight at 4°C. The membranes were washed with TBS-T three times and incubated with secondary antibodies for 2 h at 37°C. *β*-Actin and GAPDH served as the internal controls. Finally, the protein bands were visualized using the ChemiDoc XRS Gel Image system and analyzed with the Image Lab software (version 3.0; Bio-Rad Laboratories, Hercules, CA, USA).

### 2.11. Statistical Analysis of Data

Data were expressed as the mean ± standard deviation (SD) and statistically analyzed using GraphPad Prism 6.0; one-way ANOVA and Newman-Keuls were used for the comparison between the groups. *P* < 0.05 was statistically significant.

## 3. Results

### 3.1. The Changes of NLRP3 Inflammasome Protein Expression in HG-Treated H9C2 Cells with Different Intervention Time

**Figure 1:**
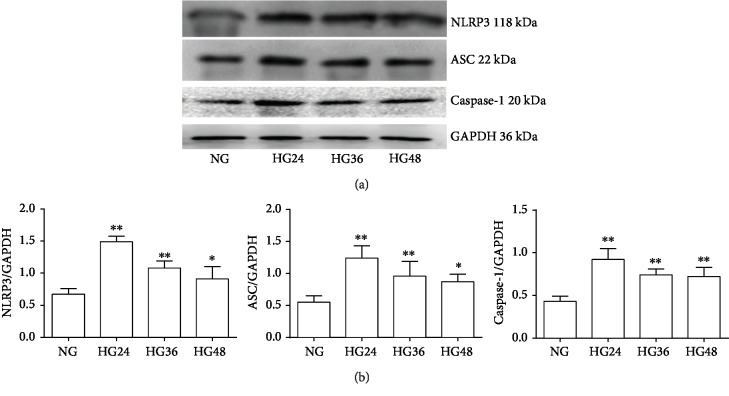
The changes of NLRP3, ASC, and Caspase-1 at protein levels after the H9C2 cardiac cells were treated with high glucose for 24, 36, and 48 hours (mean ± SD, *n* = 4). ^∗^*P* < 0.05 and ^∗∗^*P* < 0.01*vs.* NG. (a) Representative western blots of NLRP3, ASC, and Caspase-1 and GAPDH protein expression in H9C2 cardiac cells. (b) NLRP3, ASC, and Caspase-1 protein levels in H9C2 cardiac cells normalized by GAPDH levels.

Firstly, we examined the effect of HG with different intervention time in cardiac cells. In contrast to the NG group, with the time prolongation, the NLRP3 inflammasome key components—NLRP3, ASC, and Caspase-1 protein expression levels—were all increased in the H9C2 cardiac cells at 24 h, 36 h, and 48 h after treatment with 35 mM glucose (*P* < 0.01), and the highest time point is 24 h, then decreasing gradually at 36 h and 48 h (Figure 1). So we selected HG treatment for 24 h as the intervention time in the latter experiment.

### 3.2. Successful Construction of ALDH2 Gene Overexpression in H9C2 Cell Line

**Figure 2:**
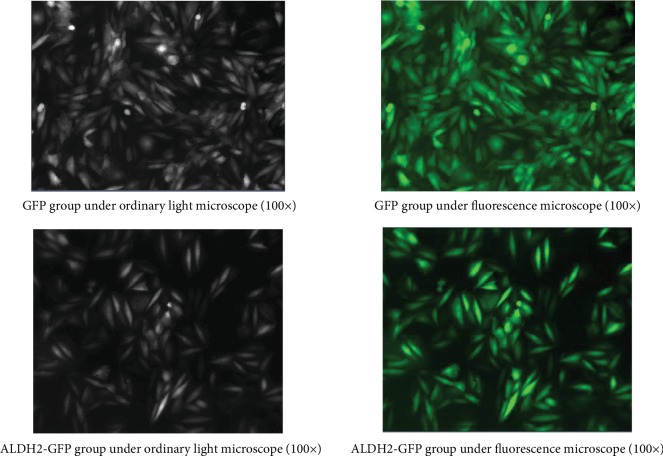
Features of H9C2 cardiac cells under optical microscopy (100x).

Since the lentivirus carries the green fluorescence gene, when they transferred into H9C2 cells, we observed the cells screened by puromycin had a green fluorescence transfection efficiency of more than 95% under the fluorescence microscope (Figure 2).

### 3.3. Changes of ALDH2 Protein and mRNA Levels in ALDH2 Overexpression H9C2 Cell Line

In the sequence of the ALDH2 gene overexpressing lentivirus vector—Ubi-MCS-3FLAG-SV40-EGFP-IRES-puromycin, the target gene fusion protein is about 59 kDa, and the target gene is fused with a 3x Flag tag, which is about 2.7 kDa. Therefore, the fusion protein is slightly larger than the background protein. Through western blot measurement, a characteristic band near 59 kDa can be observed, and its size is consistent with ALDH2 fusion protein.

**Figure 3:**
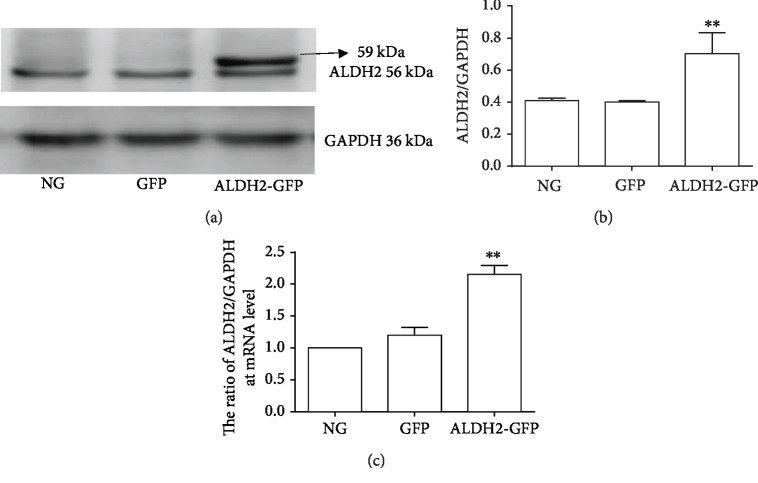
The expression of ALDH2 at protein and mRNA levels in H9C2 cardiac cells after transfection (mean ± SD, *n* = 3). ^∗∗^*P* < 0.01*vs.* NG. (a) Typical western blot bands of ALDH2 protein expression in H9C2 cardiac cells. (b) The ratio of ALDH2/GAPDH at protein level. (c) ALDH2 mRNA levels in H9C2 cardiac cells normalized by GAPDH levels.

There was no statistical difference in the expression of ALDH2 mRNA and protein levels between the GFP and NG groups. The expressions of ALDH2 at mRNA and protein levels in the ALDH2-GFP group were significantly higher than those in the GFP group (*P* < 0.01) (Figure 3). The results showed that ALDH2 overexpression in H9C2 cell was constructed successfully.

### 3.4. Changes of Cell Viability

There were no significant changes in the cell viability among the GFP, ALDH2-GFP, and NG groups, suggesting GFP and ALDH2 overexpression had no effect on cell viability in normal situation, so the GFP and ALDH2-GFP groups were not done in the latter experiments.

**Figure 4:**
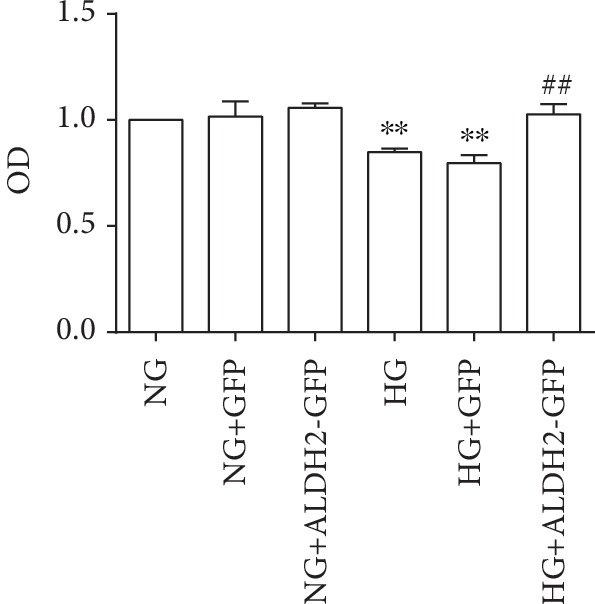
The cell viability of H9C2 cardiac cells in the different groups (mean ± SD, *n* = 5). ^∗∗^*P* < 0.01*vs.* NG; ^##^*P* < 0.01*vs.* HG and HG+GFP.

In contrast to that in the NG group, the cell viabilities in the HG and HG+GFP groups were decreased. Compared with that in the HG group, the cell viability in the HG+ALDH2-GFP group was increased (*P* < 0.01) (Figure 4), suggesting that ALDH2 overexpression increased cell viability in HG condition.

### 3.5. Changes of Mitochondrial ALDH2 Activity and Protein Expression

**Figure 5:**
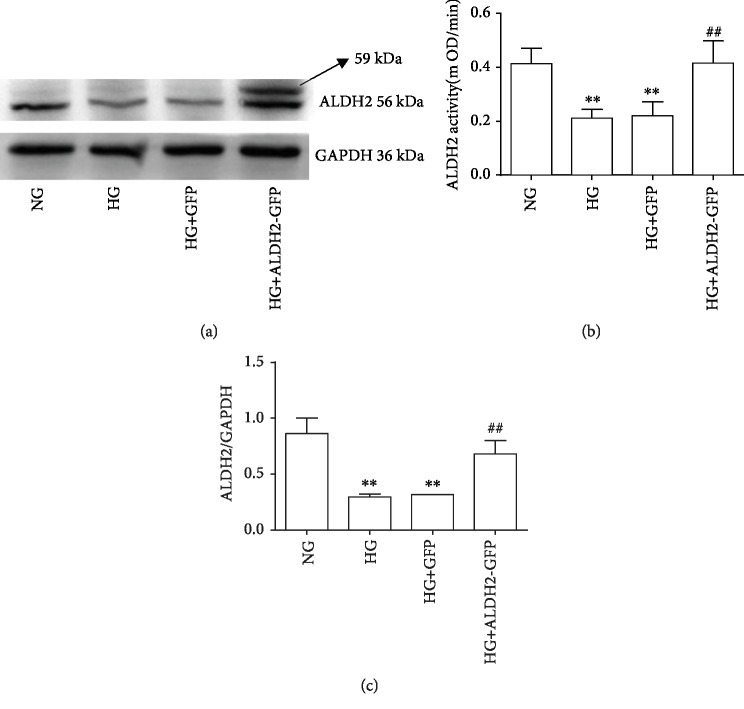
Changes of mitochondrial ALDH2 activity and protein levels in H9C2 cardiac cells (mean ± SD, *n* = 4). ^∗∗^*P* < 0.01*vs.* NG; ^##^*P* < 0.01*vs.* HG and HG+GFP. (a) Typical western blot bands of ALDH2 protein expression. (b) The result of ALDH2 activity. (c) The ratio of ALDH2/GAPDH.

From Figure 5, the results showed that mitochondrial ALDH2 activity and protein expression were significantly decreased in the HG and HG+GFP groups compared with the NG group (*P* < 0.01). Compared with the HG group, mitochondrial ALDH2 activity and protein expression were increased in the HG+ALDH2-GFP group (*P* < 0.01).

### 3.6. Changes of 4-HNE Level of in Supernatants

**Figure 6:**
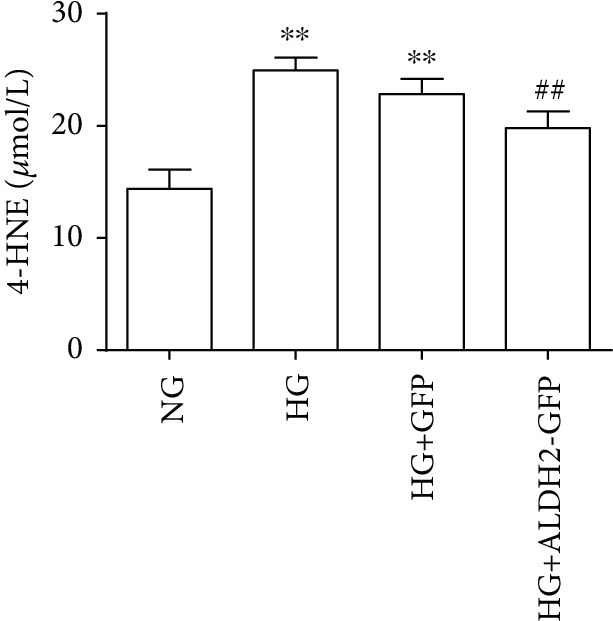
The level of 4-HNE after high glucose intervention (mean ± SD, *n* = 5). ^∗∗^*P* < 0.01*vs.* NG; ^##^*P* < 0.01*vs.* HG and HG+GFP.

Compared with that in the NG group, the 4-HNE level in the supernatant in the HG and HG+GFP groups was increased (*P* < 0.01); compared with that in the HG and HG+GFP groups, the 4-HNE level in the HG+ALDH2-GFP group was decreased (*P* < 0.01) (Figure 6) .

### 3.7. Changes of Mitochondrial Oxidative Stress by Mitosox Reagent Measurement

**Figure 7:**
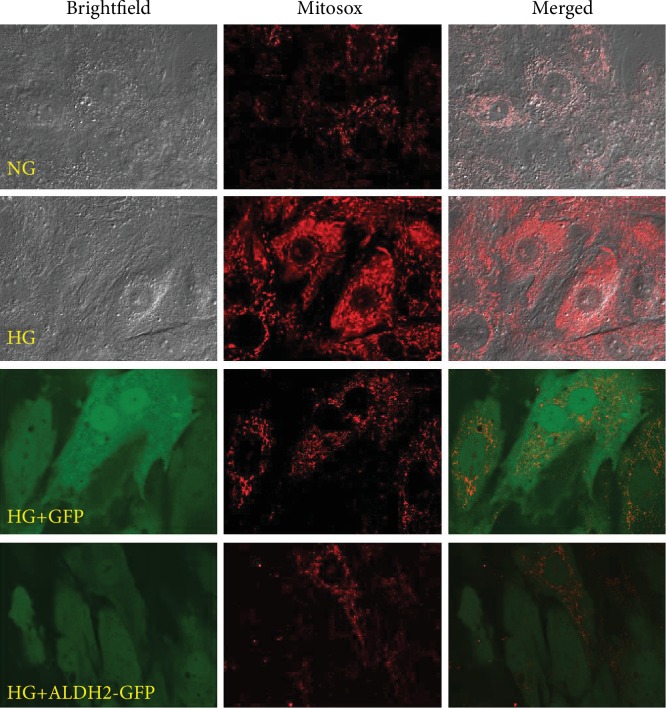
Detection of mitochondrial oxidative stress by Mitosox fluorescence staining in H9C2 cardiac cells (oil immersion lens: 600x).

Mitosox, a superoxide red fluorescent probe that specifically targets mitochondria, can selectively detect the superoxide content in the mitochondria, thereby reflecting the extent of mitochondrial oxidative stress. From Figure 7, the results showed that compared with those in the NG group, the red fluorescence intensities in HG and HG+GFP groups were increased, suggesting that mitochondrial oxidative stress level was increased, and the red fluorescence intensity in the HG+ALDH2-GFP group was significantly weakened compared with those in the HG and HG+GFP groups, suggesting that the mitochondrial oxidative stress level was decreased.

### 3.8. Changes of NLRP3, ASC, Caspase-1, and IL-18 Protein Expression

**Figure 8:**
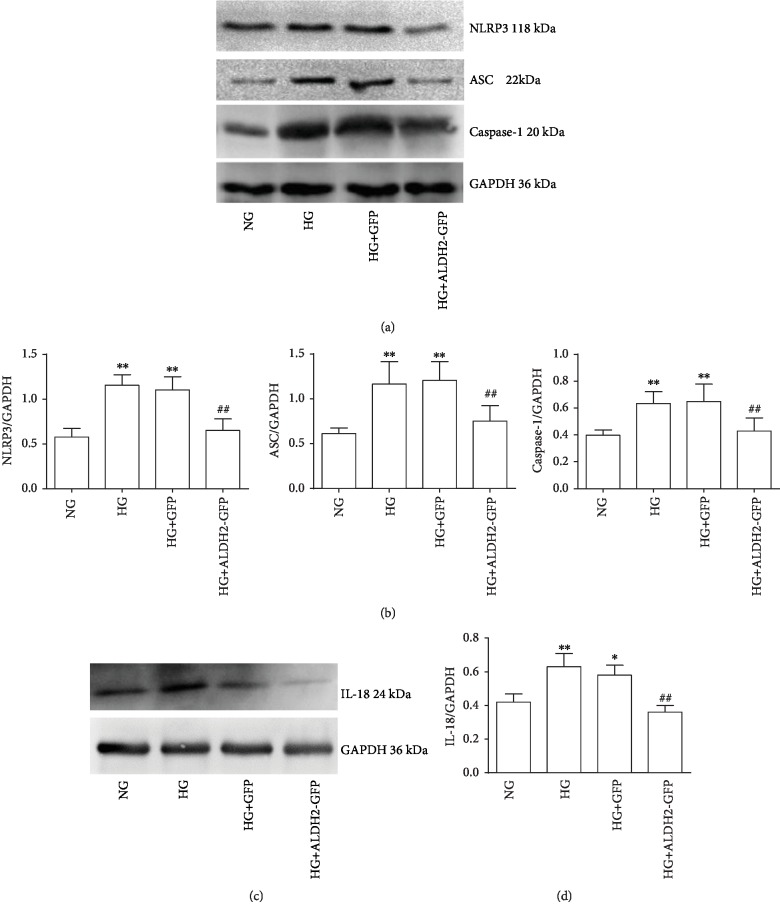
Changes in NLRP3, ASC, Caspase-1, and IL-18 protein levels in each group. (a) Representative blots of NLRP3, ASC, Caspase-1, and GAPDH in H9C2 cardiac cells. (b) NLRP3, ASC, and Caspase-1 protein levels in H9C2 cardiac cells normalized by GAPDH levels. (c) Representative blots of IL-18 and GAPDH in H9C2 cardiac cells. (d) IL-18 protein levels in H9C2 cardiac cells normalized by GAPDH levels (mean ± SD, *n* = 4). ^∗^*P* < 0.05 and ^∗∗^*P* < 0.01*vs.* NG; ^##^*P* < 0.01*vs.* HG and HG+GFP.

The western blot results showed that NLRP3, ASC, Caspase-1, and IL-18 protein expressions in the HG and HG+GFP groups were increased compared with those in the NG group. The levels of NLRP3, ASC, Caspase-1, and IL-18 protein expressions in the HG+ALDH2-GFP group were significantly lower than those in the HG group (*P* < 0.01) (Figure 8), suggesting overexpression of ALDH2 can inhibit the occurrence of NLRP3 inflammasome and IL-18 expression.

### 3.9. Changes of IL-18 and Caspase-3 Protein Expression

**Figure 9:**
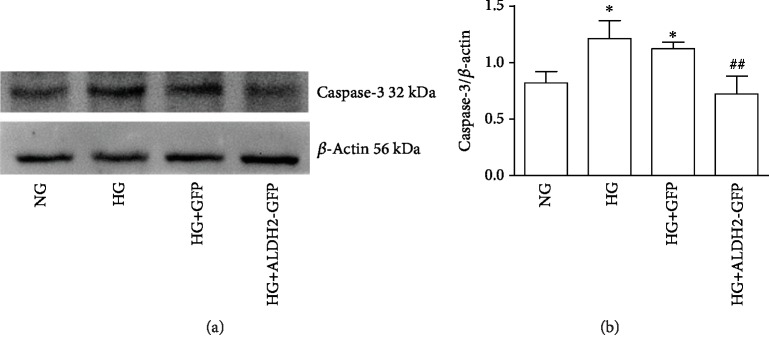
Changes of Caspase-3 protein level in each group. (a) Representative blots of Caspase-3 in H9C2 cardiac cells. (b) Caspase-3 normalized by *β*-actin level protein levels in H9C2 cardiac cells (mean ± SD, *n* = 4). ^∗^*P* < 0.05*vs.* NG; ^##^*P* < 0.01*vs.* HG and HG+GFP.

The results showed that IL-18 and Caspase-3 protein expressions in the HG and HG+GFP group were increased compared with those in the NG group. In contrast to those in the HG group, the levels of IL-18 and Caspase-3 protein expressions in the HG+ALDH2-GFP group were lower (*P* < 0.01) (Figure 9), suggesting apoptosis also participated in the HG-induced cell injury; overexpression of ALDH2 can inhibit the occurrence of apoptosis and also inhibit IL-18 expression.

### 3.10. Changes of LDH, IL-18, and IL-1*β* Levels in Supernatants

**Figure 10:**
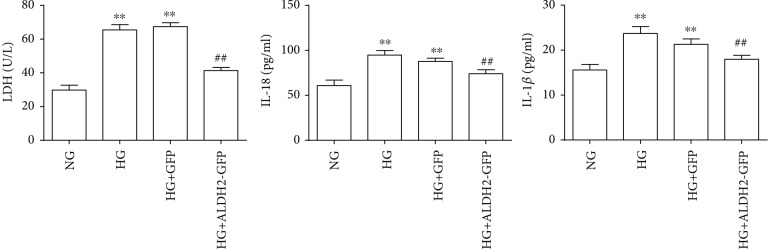
Levels of LDH, IL-18, and IL-1*β* after high glucose intervention (mean ± SD, *n* = 5). ^∗∗^*P* < 0.01*vs.* NG; ^##^*P* < 0.01*vs.* HG and HG+GFP.

The results showed that compared with those in the NG group, the levels of LDH, IL-18, and IL-1*β* in the supernatant in the HG and HG+GFP groups were significantly increased (*P* < 0.01). Compared with those in the HG group, the levels of LDH, IL-18, and IL-1*β* in the HG + ALDH2-GFP group were significantly decreased (*P* < 0.01) (Figure 10).

## 4. Discussion

In the study, we aimed to explore the effect and potential mechanisms of ALDH2 on pyroptosis (especially NLRP3 inflammasome) in high glucose-induced cardiac injury. The present study demonstrated for the first time that ALDH2 overexpression alleviated high glucose-induced H9C2 cell pyroptosis through inhibiting inflammation and mitochondria-derived ROS release; ALDH2 might play the potential role against high glucose-induced cardiac cell pyroptosis.

In the study, we transfected H9C2 cardiac cells with the lentivirus ALDH2 gene in vitro; it was found that H9C2 cardiac cells could be transfected and highly expressed by lentivirus vectors carrying ALDH2 gene successfully, and the cell activity was not affected after transfection, indicating that the transfection process did not affect the cell's function, which provided the basis for the next research.

Pyroptosis as a cell death pattern can induce reactive oxygen species (ROS) release (especially mitochondrial ROS release) and inflammation and shares some features with apoptosis, including loss of plasma membrane integrity and release of intracellular contents, such as LDH [17]. In the pathological process of cardiovascular disease, ROS overproduction causes a series of damage effects. These excessive ROS directly lead to cell mitochondrial damage, DNA damage, and intracellular energy depletion and ultimately cause the cell death [18, 19]. The mitochondrion is a major source of intracellular ROS production. Studies have shown that ROS signaling required for NLRP3 inflammasome activation is mainly derived from mitochondria, suggesting that mitochondria-derived ROS plays a key role in NLRP3 inflammasome activation [10, 11]; inhibiting mitochondrial ROS generation significantly inhibits NLRP3 inflammatory activation [20–22]. 4-HNE is a highly active lipid peroxidation end product related to oxidative stress closely. When the body was stimulated by oxidative stress, 4-HNE concentration increased rapidly [23]. 4-HNE not only produces the toxic effects but also participates in biochemical processes such as signal transduction, oxidative stress, and mediated cell death and induces the inflammatory response.

Previous studies have confirmed that mitochondrial ALDH2 has the functions of reducing oxygen free radical production, scavenging aldehydes and 4-HNE, and finally protecting mitochondrial function [24, 25]. Zhang et al. [26] observed that activation of ALDH2 protects rats from hepatic ischemia/reperfusion injury by inhibiting oxidative stress and clearing the accumulation of 4-HNE. Our previous study had found in diabetic rats that ALDH2 reduced oxidative stress, 4-HNE content, and IL-1 level [27]. In our study, after intervention of 35 mM glucose for 24 h, we observed that with the decrease of cell viability, LDH, 4-HNE levels and mitochondrial ROS release were increased; after overexpression of ALDH2, the cell viability was increased, LDH, 4-HNE levels and mitochondrial ROS release were decreased, suggesting mitochondrial ALDH2 can eliminate LDH and 4-HNE accumulation and reduce the production of mitochondrial oxygen free radicals.

Hyperglycemia not only induced excessive mitochondrial ROS production but also induced the inflammatory reactions [8, 9]. The previous studies have shown that NLRP3 inflammasome-related pyroptosis plays a vital role in the development of diabetic cardiomyopathy [28, 29]. During pyroptosis, when NLRP3 inflammasome was activated, NLRP3 induced the self-polymerization and recruitment of ASC, which activating pro-caspase-1 into caspase-1, caspase-1 cleaved pro-IL-1*β* and pro-IL-18 into their active forms, IL-1*β* and IL-18. When IL-1*β* and IL-18 were released to the outside of the cell, the inflammatory process was amplified; it caused the explosive local and systemic inflammatory factors' secretion and induced the damage of tissue and organ [30, 31]. However, the mechanism of NLRP3 inflammasome activation in HG condition remains to be explored. Whether ALDH2 can alleviate the occurrence of high glucose-induced pyroptosis has not been reported. Our results showed that after high glucose intervention, NLRP3, ASC, caspase-1, and IL-18 expressions, IL-1*β* and IL-18 levels were all increased, when ALDH2 was overexpressed, NLRP3, ASC, caspase-1, and IL-18 protein expression levels were reduced, IL-1*β* and IL-18 levels in supernatants were also reduced, indicating that ALDH2 could reduce high glucose-induced NLRP3 inflammasome activation and pyroptosis.

In the study, we also observed that Caspase-3 protein expression was also increased in HG condition and, when ALDH2 was overexpressed, Caspase-3 protein expression was attenuated, suggesting that ALDH2 overexpression also inhibited apoptosis. The proportion and relationship of different cell death types were worthy of intensive investigation.

There are some limitations in the study. We only observed the changes of NLRP3 inflammasome, inflammatory factors, and mitochondrial ROS after overexpression ALDH2, not to investigate what changes if NLRP3 inflammasome or mitochondrial ROS were inhibited. In the future, we will investigate the intensive mechanisms.

In conclusion, in our study, ALDH2 can protect the H9C2 cardiac cells against hyperglycemia-induced inflammation by suppressing the production of mitochondrial oxygen free radicals and stimulating NLRP3 inflammasome activation. This provides an important basis for clarifying the likely mechanisms in high glucose-induced cardiac cell injury.

## Data Availability

The data used to support the findings of this study are available from the corresponding authors upon request.
