# Corrigendum to “ALDH2 Overexpression Alleviates High Glucose-Induced Cardiotoxicity by Inhibiting NLRP3 Inflammasome Activation”

**DOI:** 10.1155/2021/9813687

**Published:** 2021-08-05

**Authors:** Ruiping Cao, Dian Fang, Jiahui Wang, Ying Yu, Hongwei Ye, Pinfang Kang, Zhenghong Li, Hongju Wang, Qin Gao

**Affiliations:** ^1^Department of Physiology, Bengbu Medical College, Bengbu, Anhui 233030, China; ^2^Department of Cardiovascular Disease, The First Affiliated Hospital of Bengbu Medical College, Bengbu, Anhui 233004, China

In the article titled “ALDH2 Overexpression Alleviates High Glucose-Induced Cardiotoxicity by Inhibiting NLRP3 Inflammasome Activation” [1], the authors Ruiping Cao, Dian Fang, Jiahui Wang, Ying Yu, Hongwei Ye, Zhenghong Li, and Qin Gao were affiliated to the Department of Physiology, Bengbu, Anhui, 233030, China which is incorrect.

The correct affiliation for the authors is: Department of Physiology, Bengbu Medical College, Bengbu, Anhui, 233030, China.
